# Advanced quantitative evaluation of PET systems using the ACR phantom and NiftyPET software

**DOI:** 10.1002/mp.15596

**Published:** 2022-03-31

**Authors:** Pawel J. Markiewicz, Casper da Costa‐Luis, J. Dickson, A. Barnes, G. Krokos, J. MacKewn, T. Clark, C. Wimberley, G. MacNaught, M. M. Yaqub, J. D. Gispert, B. F. Hutton, P. Marsden, A. Hammers, A. J. Reader, S. Ourselin, K. Herholz, J. C. Matthews, F. Barkhof

**Affiliations:** ^1^ Centre for Medical Image Computing Department of Medical Physics and Biomedical Engineering University College London London UK; ^2^ School of Biomedical Engineering and Imaging Sciences King's College London UK; ^3^ Institute of Nuclear Medicine University College London Hospitals London UK; ^4^ Edinburgh Imaging The University of Edinburgh Edinburgh UK; ^5^ Department of Radiology and Nuclear Medicine Amsterdam UMC Vrije Universiteit Amsterdam Netherlands; ^6^ Barcelonaβeta Brain Research Center (BBRC) Pasqual Maragall Foundation Barcelona Spain; ^7^ Institute of Nuclear Medicine University College London London UK; ^8^ Division of Neuroscience & Experimental Psychology University of Manchester UK; ^9^ Sheffield Institute for Translational Neuroscience University of Sheffield Sheffield UK

**Keywords:** analysis, MR, PET, phantom, precision, registration, resolution

## Abstract

**Purpose:**

A novel phantom‐imaging platform, a set of software tools, for automated and high‐precision imaging of the American College of Radiology (ACR) positron emission tomography (PET) phantom for PET/magnetic resonance (PET/MR) and PET/computed tomography (PET/CT) systems is proposed.

**Methods:**

The key feature of this platform is the vector graphics design that facilitates the automated measurement of the knife‐edge response function and hence image resolution, using composite volume of interest templates in a 0.5 mm resolution grid applied to all inserts of the phantom. Furthermore, the proposed platform enables the generation of an accurate μ‐map for PET/MR systems with a robust alignment based on two‐stage image registration using specifically designed PET templates. The proposed platform is based on the open‐source *NiftyPET* software package used to generate multiple list‐mode data bootstrap realizations and image reconstructions to determine the precision of the two‐stage registration and any image‐derived statistics. For all the analyses, iterative image reconstruction was employed with and without modeled shift‐invariant point spread function and with varying iterations of the ordered subsets expectation maximization (OSEM) algorithm. The impact of the activity outside the field of view (FOV) was assessed using two acquisitions of 30 min each, with and without the activity outside the FOV.

**Results:**

The utility of the platform has been demonstrated by providing a standard and an advanced phantom analysis including the estimation of spatial resolution using all cylindrical inserts. In the imaging planes close to the edge of the axial FOV, we observed deterioration in the quantitative accuracy, reduced resolution (FWHM increased by 1–2 mm), reduced contrast, and background uniformity due to the activity outside the FOV. Although it slows convergence, the PSF reconstruction had a positive impact on resolution and contrast recovery, but the degree of improvement depended on the regions. The uncertainty analysis based on bootstrap resampling of raw PET data indicated high precision of the two‐stage registration.

**Conclusions:**

We demonstrated that phantom imaging using the proposed methodology with the metric of spatial resolution and multiple bootstrap realizations may be helpful in more accurate evaluation of PET systems as well as in facilitating fine tuning for optimal imaging parameters in PET/MR and PET/CT clinical research studies.

## 1 INTRODUCTION

Phantoms are test objects that provide an accurate means of calibration and evaluation of the performance of imaging systems such as positron emission tomography (PET).^1^ Phantoms are helpful in the standardization and assessment of the reproducibility and variability of PET performance, which is important for multicenter clinical studies^2^, ^3^, ^4^, ^5^, ^6^ and in optimization of imaging protocols, including image reconstruction methods.^7^, ^8^, ^9^, ^10^, ^11^, ^12^

Phantom imaging is subject to limited shapes and constrained reliability of filling and imaging.^13^ Nevertheless, simple phantom shapes (such as spheres and cylinders) are more suitable for evaluation and harmonization of PET systems in multisite settings, particularly when used in conjunction with automated analyses of image data.^14^, ^15^, ^16^, ^17^ However, such phantoms are difficult to image with PET/MR systems. Large water compartments often induce MR artifacts, and the acrylic housing of phantoms produce weak MR signal,^18^ making accurate attenuation correction challenging.^19^ Furthermore, one of the most important factors affecting PET image performance is spatial resolution—which is difficult to measure directly using phantoms.^20^

In this work, we propose a novel platform composed of software tools optimized for automated PET/MR phantom imaging (also applicable to PET/CT systems). In addition to the standard analyses, the platform offers direct estimation of effective spatial resolution, as well as accurate attenuation correction using specifically designed high definition templates and robust image registration. The Jaszczak Deluxe Flangeless ECT phantom with a modified faceplate for PET, approved by the American College of Radiology (ACR), has been selected for this work as it offers multiple tests with many hot and cold inserts,^2^, ^14^ useful in simulating hyper‐ and hypometabolism present in dementia and epilepsy imaging. The shape of the different cylindrical inserts of the phantom enables accurate extraction of the edge response function using concentric ring sampling, based on which spatial resolution can be quantitatively evaluated. As there are many possible configurations of the phantom, it is difficult to justify the use of standard PET/MR attenuation corrections. Therefore this work aims to fill this gap for automated analysis software with additional advanced metrics for PET/MR and PET/CT. The proposed software platform uses the open‐source Python package *NiftyPET*,^21^ which facilitates straightforward experimentation with all image reconstruction parameters as well as generation of multiple bootstrap realizations of the list‐mode data for the assessment of uncertainty of all image metrics. The utility of the platform is demonstrated in the evaluation of the impact of the activity outside the field of view (FOV) and image reconstruction with and without the incorporated PSF. We intend to use this phantom and proposed analysis in the harmonization of the Dementias Platform UK (DPUK) network, consisting of eight sites equipped with Siemens Biograph mMR and GE Signa PET/MR scanners.

## 2 METHODS

### 2.1 PET data acquisition with and without outside FOV activity

The ACR‐approved Jaszczak Deluxe Flangeless ECT phantom with an Esser faceplate for PET (referred to here as the “ACR phantom”) was used here. The phantom spheres, which are used in SPECT imaging, were removed and all the compartments were filled according to the ACR instructions using PET tracer [${}^{18}$F]FDG with two separate activity solutions (doses) for the inserts and background compartments, both measured 1 h before acquisition. The doses were 8.3 MBq (diluted in 1 L) for the inserts and 18.7 MBq (diluted in approximately 5.7 L) for the background, resulting in a ratio of 2.5:1 and corresponding to a simulated patient dose of approximately 200 MBq. The phantom was scanned in a PET/MR Siemens Biograph mMR scanner, in two sessions of 30 min each, without moving the bed such that for the second acquisition, an external activity source (184 MBq, measured at 1 h before scan start) was added just outside the axial FOV. The external activity in the second scan was contained in a uniform cylinder, 30 cm long and ⌀20 cm, placed next to the ACR phantom faceplate, mainly affecting the reconstructed activity of the cold and hot inserts and the nearby background.

**FIGURE 1:**
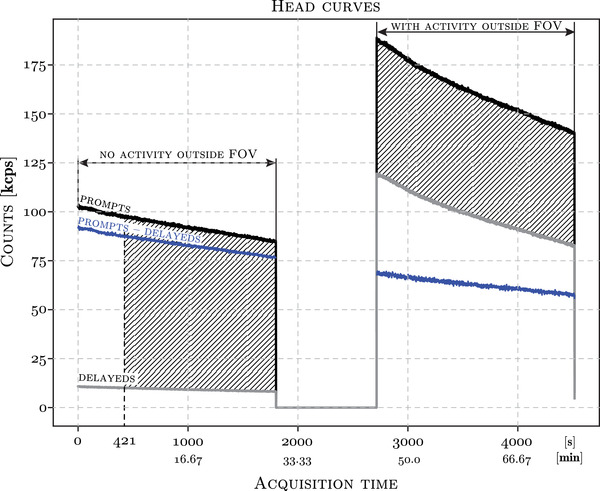
Total acquired prompt (black) and delayed (gray) events versus time, and their difference (blue), for the two consecutive 30 min acquisitions of the ACR phantom, with and without the activity outside the FOV

The total acquired prompt and delayed events per second for the two acquisitions, at exactly the same table position, are shown in Figure 1. In order to compare the two image reconstructions corresponding to the two acquisitions with and without the activity outside FOV, the overall prompts reduced by the delayed events were equal for both scans (the shaded areas for both acquisitions in Figure 1 are approximately equal). Hence, scan times were adjusted as to have similar count levels for both scans, while accounting for the decay and increased random events for the later scan. The start time of the first acquisition was delayed by 421 s (7 min), while the second scan was left intact (30 min). For image registration purposes, the first full acquisition (30 min) was used. The doses and acquisition times used for the phantom imaging were chosen so as to simulate those used in amyloid imaging with [${}^{18}$F]flutemetamol—approximately 185 MBq and 20 min scan times. All the processing of list‐mode data were performed using the *NiftyPET* package.^22^ After the two acquisitions were finished, two samples from the hot inserts and the background were taken for well counter measurements for gold standard activity concentration references.

The proposed open‐source platform for image analysis of the PET version of the ACR Jaszczak phantom is based on high‐definition template designs and the *NiftyPET* Python package for image reconstruction and analysis. Such an approach enables full control over the reconstruction process with precise PET signal extraction using spatially granular volume of interests (VOIs). The high precision is achieved by robust alignment of sampling VOIs in the PET space using PET templates specifically designed for image registration. Such robust alignment of specially designed concentric VOIs facilitates more sensitive image analysis for better detection of abnormalities compared to manually placed and/or drawn VOIs. The cylindrical phantom is made up of acrylic glass with the internal diameter of 210 mm and height of 190 mm, and consists of three main parts: (1) faceplate with four hot inserts of variable diameter (⌀25 mm, ⌀16 mm, ⌀12 mm, ⌀8 mm), two cold cylinders of ⌀25 mm containing air and water, and a ⌀25 mm Teflon cylinder simulating bone (all the inserts, apart from the Teflon cylinder, have a surrounding 1.5 mm thick wall); (2) a region of uniformity in the middle; and (3) acrylic resolution rods arranged as six sets of uniformly spaced rods with different diameters: 4.8, 6.4, 7.9, 9.5, 11.1, and 12.7 mm.

### 2.2 Digital template design

**FIGURE 2:**
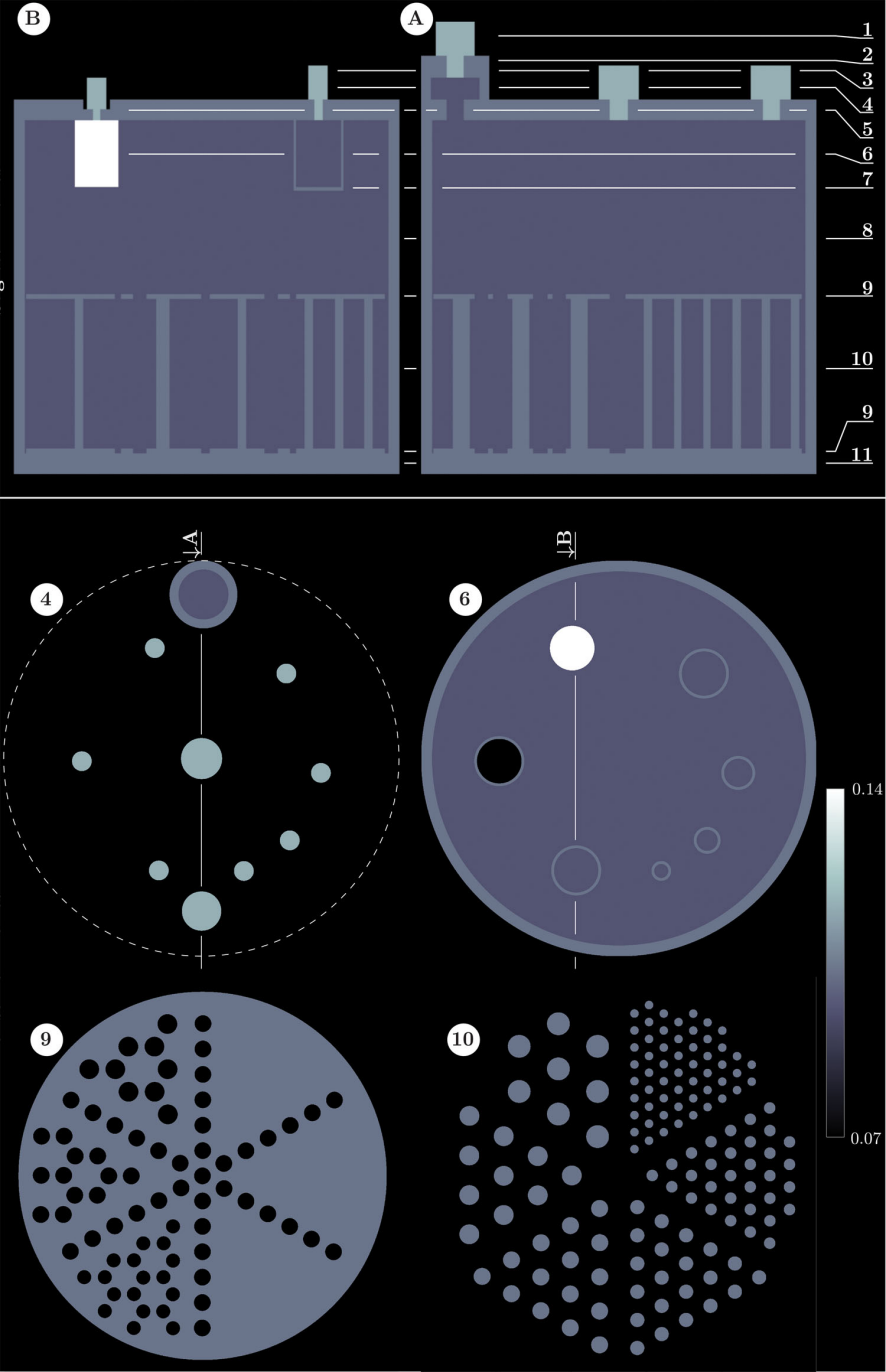
The 3D design of the PET version of the Jaszczak ACR phantom based on vector graphics representations of each unique transaxial section profile of the phantom (numbered from 1 to 11). Two sagittal views are shown (*A* and *B*) as marked on the transaxial section profiles 4 and 6 below. Note that the resolution rod insert (sections 9 and 10) are designed separately as they can be fitted in many possible ways within the phantom. All compartments are shown in terms of PET μ‐values of the acrylic material and water

The 3D shape of the ACR Jaszczak phantom with all its inserts and screws is fully represented using eleven 2D transaxial sectional profiles (Figure 2) laid out in *Adobe Illustrator* using vector graphics with multiple layers (these will be available as open source at https://niftypet.readthedocs.io). The dimensions for all components were taken from the nominal expected values and confirmed with physical measurements and a high‐resolution CT scan of the actual phantom used to acquire data in this work. The 2D sectional profiles of the phantom are numbered from 1 to 11 (Panels A and B in Figure 2) with the key transaxial sections shown in the lower part of the figure. All the components are accurately represented, including the acrylic insert walls, 1.5 mm thick (section #6), as well as all the rods of the lower part of the phantom (sections #9 and #10). The resolution rod component of the phantom is designed as a separate part of the phantom and independently aligned to the PET data as there are infinite positions (rotations and flips) governing how the rods are placed within the main phantom compartment. The image intensities shown in the design represent the linear attenuation coefficient μ for 511 keV gamma rays. The exact values of the attenuation coefficient for acrylic components were based on specific measurement using a PET camera^23^ and confirmed using a CT scan followed by a piecewise linear transformation of the Hounsfield units (HUs) to PET μ‐values [cm${}^{-1}$].^24^ The μ‐values used for the water, nylon screws, acrylic, and the Teflon cylinder were 0.096, 0.1036, 0.117, and 0.148 cm${}^{-1}$, respectively. All the unique transaxial section profiles were exported to high‐resolution (300 DPI) PNG graphics files, read in Python and downsampled to voxel size of 0.2 mm. These were then arranged together over a number of axial slices corresponding to the axial dimensions for each unique section, thus forming a fully 3D μ‐map image of the phantom. The resolution rod component was designed and kept separately.

### 2.3 Two‐stage image registration

**FIGURE 3:**
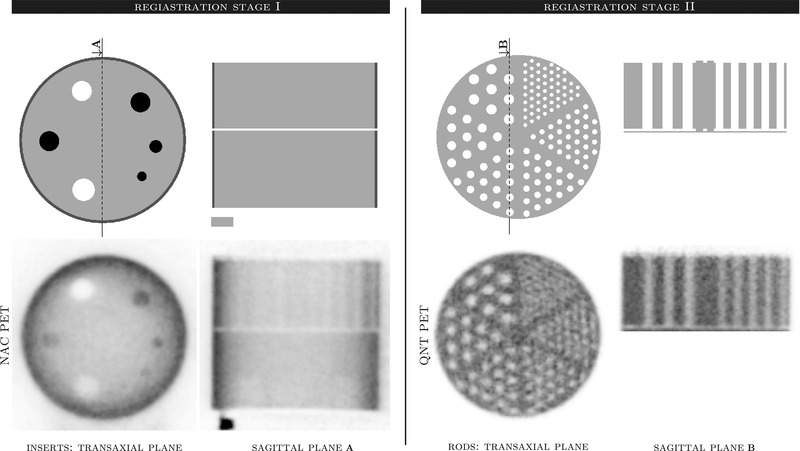
Two‐stage registration: STAGE I (left) for the whole phantom without rod insert and STAGE II (right) for the rod insert. The top rows show the NAC and QNT PET templates used only for registration. STAGE I uses the NAC PET image, while STAGE II uses the quantitative (QNT) PET (bottom rows)

The generated μ‐map must be accurately aligned with the acquired PET data to perform attenuation correction. Although the generated μ‐map could be used for the first stage (STAGE I) of image registration to the nonattenuation‐corrected (NAC) PET image, a separate template representing the NAC reconstructed image was used. The NAC PET image is distinctly different from the attenuation‐corrected image and is specific to the phantom; hence, the NAC template may yield more robust registration. The generated NAC PET template for STAGE I image registration in transaxial and sagittal views is shown in Figure 3 (left panel) alongside the corresponding reconstructed NAC image. It has to be ensured that the template of the μ‐map and the NAC PET template are in the same space, such that the transformation found through registration of the NAC template to the NAC PET image can also be applied to the μ‐map and sampling templates (see Section 2.4). To reduce the time for performing the registration, the voxel size of the templates has been reduced to 0.4 mm and the NAC PET images are cropped and upsampled from 2 to 0.5 mm isotropically. The reason for this higher resolution image registration is to ensure precise alignment particularly for all the insert areas. The rigid‐body registration was performed using the open‐source Python package *DIPY* ^25^ with the mutual information as a similarity metric and multiresolution strategy using a Gaussian pyramid (using standard deviations of 3.0, 1.0, and 0.0, with 10000, 1000, and 100 iterations for the three stages of the pyramid, respectively).

The rigid‐body transformation obtained from the registration was used to resample the μ‐map template (with 0.2 mm isotropic voxel size) to the native PET space with a voxel size of 2 mm^1^. This μ‐map, purposely without the resolution rods insert (i.e., with the μ‐values of water instead of the rods), was used for the first quantitative image reconstruction used in STAGE II registration of the resolution rod insert. Since the μ‐value for the acrylic rods is slightly higher than that of water (i.e., $\mu_{a}=0.1036$ cm${}^{-1}$ compared to $\mu_{w}=0.096$ cm${}^{-1}$), the resulting cold regions of the rods are of slightly lower values—producing artificially better contrast, and hence, potentially more accurate image registration for the rod insert.

The reconstructed PET image was further processed for the second‐stage registration by automatically detecting the resolution rods part using summed axial image profiles, in which the two end discs supporting the rods are clearly marked. The remaining components of the phantom were removed by setting their voxel intensities to zero as shown in Figure 3 (right panel). This ensures more accurate image registration of the resolution rods template to this configurable part of the phantom. Note that this insert can be placed in many possible rotations that are difficult for registration algorithms to deal with, and hence the template has to be rotated to as close a position as possible to allow the pie‐shaped rods to be accordingly registered (this is controlled by a single angular input value). The PET images as shown in Figure 3 were used only for registration purposes and not for any image analysis. The full μ‐map image was generated by resampling the core and resolution rods templates using the two separate rigid body transformations and combining the resampled μ‐maps into one μ‐map, used for a fully quantitative image reconstruction suitable for image analysis. Similar to the work of^1^ on torso phantoms, the proposed method utilizes only the PET data for alignment, as the quality of MR images for the phantoms, even with diluted additives, was not satisfactory. Furthermore, there are small but significant shifts possible between the PET and MR spaces (reported to be around 1 mm for some PET/CT systems^26^), further complicating the use of MR images for registration.

### 2.4 Sampling templates

**FIGURE 4:**
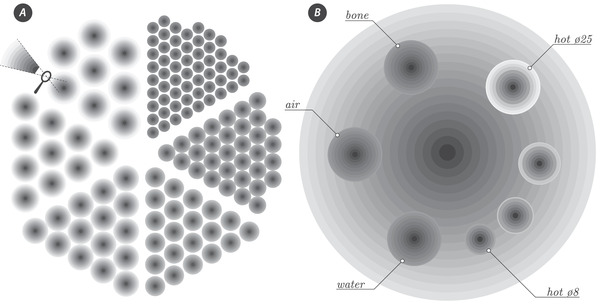
Transaxial sampling templates consisting of concentric ring (tubes in 3D) with 2 mm spacing for granular sampling of VOIs around the rods of different diameter (*A*) as well as the fillable inserts and the bone insert immersed in the uniform background (*B*), which is sampled with big concentric tubes from the center to the edge of the phantom. The hot inserts have different diameters, with the biggest ⌀25 mm and smallest ⌀8 mm inserts marked in *B*

Apart from the NAC registration and μ‐map templates, the proposed design offers a unique set of VOI templates for granular sampling of the PET image in a high‐resolution image grid (0.5 mm isotropically). Combined with the robust image registration of the templates to the PET space, a high‐precision analysis of the phantom image data is achieved as demonstrated below using bootstrap resampling. As all the templates originate in the same design space with the same voxel size, two single registrations for the two parts of phantom are sufficient, further ensuring consistency between attenuation correction and postreconstruction image analysis. The transaxial views of the sampling templates for the resolution rods and the faceplate with inserts are shown in Figure 4. The sampling pattern for each rod, insert, and the background is performed using concentric rings, which in 3D, form concentric tubes with the axis parallel to the phantom axis. The sampling of resolution rods (Figure 4 *A*) is performed using the same rings for all rods in each pie‐shaped region, regardless of the size of the rods. The sampling center is made up of a circle of ⌀2.52 mm (a tube in 3D) and is followed by a set of concentric rings with approximately 2 mm spacing. Since the smaller rods are packed more densely, the extent to which the rings can spread is limited and hence each set of rods have a different number of sampling rings (11, 10, 9, 7, 6, and 4 from the biggest to smallest rod set).

Such granular concentric sampling allows more informative investigation of the PET signal change as a response to the knife‐edge objects—rods in the warm background. The values for each ring were then added across all rods for each set. The faceplate sampling (Figure 4 *B*) consists of sampling of the background activity using 18 large concentric rings from the transaxial center of the phantom to the phantom edge, covering the whole ⌀210 mm with 12 mm spacing. In this way, the radial background uniformity can be accurately investigated from the center of the phantom, acting as a good indicator of scatter and attenuation correction performance. The sampling pattern has necessary holes to avoid sampling the insert areas and their vicinities. All the inserts are sampled with concentric rings starting with a circle of ⌀4 mm and followed by rings with 2 mm spacing (apart from the wall borders, where the spacing is 2.5 mm in order for the sampling to be aligned with the insert walls). There are 10 rings for each hot insert and 11 for each cold insert (contrast recovery from cold areas is usually more challenging). All sampling patterns are performed using concentric rings, resulting in average values from the center of each insert or rod, through the border or wall, and well into the background.

### 2.5 Estimation of spatial resolution

The sampling described above facilitates robust extraction of the knife‐edge response function, K(x), for all the inserts and rods—the contrast of radial step functions created by the inserts and rods.^27^ Robust sampling of K(x) is possible due to the circular shapes of the insert edges, which intersect image voxels at different angles and proportions, thus enabling finer sampling of the response than possible with the normal voxel spacing. A very similar principle is used by Lodge et al. (2018)^20^ but does not require tilting the phantom since the angles are created by the cylindrical inserts themselves.

Since the point spread function (PSF) is simply a derivative of K(x),^20^, ^27^ we fitted the error function, erf(x), to the sampled K(x) using four parameters:

(1) $$K\left(x\right)=A\cdot \mathrm{erf}\left(k(x-\mu )\right)+b,$$

where erf(x) is defined as:

(2) $$\mathrm{erf}\left(x\right)=\frac{2}{\sqrt{\pi }}\int_{0}^{x}e^{-t^{2}}dt.$$

Hence, the effective (measured) PSF can be approximated by the derivative of K(x), which is a Gaussian:

(3) $$\mathrm{PSF}\left(x\right)=\frac{2Ak}{\pi }\exp\left(-k^{2}{\left(x-\mu \right)}^{2}\right),$$

for which the standard deviation is $\sigma ={\left(k\sqrt{2}\right)}^{-1}$ and $\mathrm{FWHM}=2\sigma \sqrt{2\ln2}$.

For the thinner rods, where the sampling is reduced due to the densely populated rods, the spatial resolution may be better represented by the amplitude of the Gaussian, that is, the derivative of K(x) at its inflection point. It assumed that the inflection point represents the location of the insert boundary. Analyzing the effective PSF, we extracted three quantitative parameters:

1. the maximum derivative corresponding to the highest rate of transition from background to the insert activity concentration;
2. the offset between the real insert boundaries and the image‐derived boundaries obtained from the inflection point of K(x), and
3. the width of the effective PSF as given by the full width at half maximum (FWHM) of the resulting Gaussian.

### 2.6 PET image reconstruction and sampling

All images were reconstructed using the Python *NiftyPET* package for rapid processing of the list‐mode data,^22^ randoms correction and fully 3D scatter modeling^21^ and ordered subsets expectation maximization (OSEM) reconstruction^28^ with 14 subsets and varying number of iterations ({2,4,⋯,20, n=10}), as well as with and without the PSF modeled as a shift‐invariant kernel. The PSF kernel for the Siemens Biograph mMR scanner was found based on averaged point source measurements. The vendor software for image reconstruction or corrections was not used at any point of the process.

The standard ACR analysis^2^ involved calculating SUV values in a 1‐cm thick slice at the midpoint of the faceplate inserts (see plane #6 in Figure 2) using a circular ROI inside the largest hot insert. ROIs of the same size were then used for sampling all other inserts in the faceplate. The ROI masks were easily obtained as a composite of selected concentric rings for each insert, which were automatically aligned to the PET image. The PET images for this analysis were reconstructed with variable OSEM iterations (ranging from 2 to 20, with 14 subsets) and smoothed with an isotropic Gaussian of FWHM=4 mm. For the advanced analysis of spatial resolution estimation, the reconstructed images were not filtered.

### 2.7 Uncertainty analysis using bootstrap resampling

The standard analysis of the ACR phantom can be straightforwardly performed using the provided sampling templates and will be available on our website https://niftypet.readthedocs.io, together with all the PET data and templates as open source. Here we focus on evaluation of the precision (uncertainty) of the whole processing chain of acquisition, image reconstruction, registration, and analysis using bootstrap resampling of the list‐mode data.^29^, ^30^ Note that the bootstrap resampling does not account for the variability in filling or positioning of the phantom. The two phantom scans were resampled 50 times, resulting in 100 realizations for the two acquisitions. Each realization was reconstructed using OSEM with 4, 8, and 16 iterations, with and without the PSF, resulting in six images per realization. These images were used for estimating the precision of any image‐derived statistic, that is, the average VOI values using the sampling templates registered to the images independently across all realizations. While these templates enable the standard ACR analysis in an automated fashion, the focus of this work is to facilitate more detailed and insightful analysis of quantitative PET performance using this phantom.

## 3 RESULTS

The automated analysis of the ACR phantom was performed using the two scans, with and without the activity outside the FOV, reconstructed varying iterations, as well as with the PSF included in the reconstruction (PSF‐OSEM) for the scan without the activity outside the FOV. The phantom analysis with the proposed automated image sampling was performed on 30 reconstructed images without bootstrap resampling (2×10 reconstructions for the first acquisition and 10 for the second acquisition, as the PSF reconstruction for the external activity was not considered). The uncertainty analysis of all VOI values was facilitated by 50 bootstrap realizations of both acquisitions, and for only three different OSEM iteration numbers, that is, {4,8,16}, due to considerable computing resources required for such resampling. This resulted in an additional 450 image reconstructions (3 reconstructions × # 3 OSEM iterations × 50 realizations) for the bootstrapped list‐mode datasets.

### 3.1 Sampling the faceplate inserts and background

**FIGURE 5:**
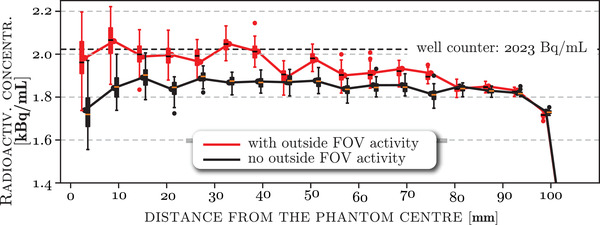
Measured mean background radioactivity concentration with no activity outside the FOV (black) and **with** activity outside the FOV (red). The concentrations are plotted for all concentric VOIs spanning the whole phantom transaxially, from the center to the edge (cf. Figure 4 *B*). The curves are found using image reconstruction of list‐mode data without resampling, while the boxes represent the distribution of 50 bootstrap realizations of the mean concentration for each concentric VOI. The well counter measurement of the background is shown with the black dashed line

The uniformity of the background was sampled around the faceplate cylindrical inserts using 18 concentric tubular VOIs, 1 cm thick axially (for the transaxial view of the concentric VOIs, see Figure 4 *B*). The circular VOIs at the center have smaller sampling volume, and hence are noisier, compared to the VOIs toward the edge of the phantom. The average radioactivity concentration across the whole background, as measured by the concentric VOIs, is shown in Figure 5 for the acquisition without (black curve and boxplots) and with the activity outside the FOV (red curve and boxplots). The well counter measurement of the background is shown by the dashed black line. The boxplots of the distribution of the mean VOI concentration across bootstrap realizations reveal larger uncertainties of central VOIs especially for the scan with activity outside the FOV. Note the increased nonuniformity toward the central part of the phantom for the scan with the added activity outside the FOV.

**FIGURE 6:**
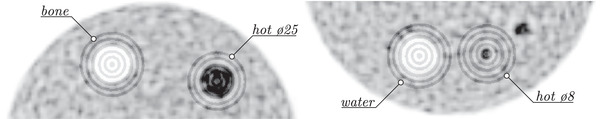
VOI sampling using concentric rings with 2 mm spacing for hot (10 rings) and cold (11 rings) inserts of the phantom faceplate. The sampling is shown for the bone and ⌀25‐mm hot inserts (left) as well as for the cold water and ⌀8‐mm hot inserts (right). Note the higher image resolution grid required for this spatially precise sampling

The sampling of the faceplate inserts using concentric VOIs is shown in Figure 6 by superimposing the concentric VOIs on the reconstructed PET images of some of the cold and hot inserts. The extracted mean VOI values with uncertainty estimation using bootstrap resampling for all the reconstructions are shown in the Supporting Information (see Section S2). The standard phantom analysis using summed ring VOIs and following the ACR accreditation criteria has also been presented in the Supporting Information (see Section S1).

### 3.2 Sampling the resolution rods

**FIGURE 7:**
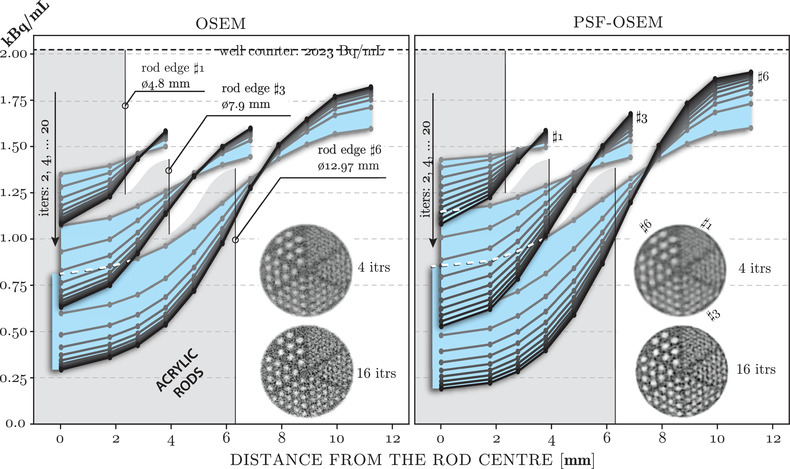
Radioactivity concentration curves for the three sets of acrylic rods with diameters of ⌀4.8 mm (#1), ⌀7.9 mm (#3), and ⌀12.97 mm (#6), respectively. The curves were obtained by sampling the PET images with concentric ROI rings (cf. Figure 4 *A*). The curves are shown for OSEM (left) and PSF‐OSEM (right) in the reconstruction. A range of OSEM iterations were considered: 2,4,⋯,20 marked with colors ranging from gray to black, respectively. In each plot superimposed are the images of 1 cm slice thickness for each reconstruction with 4 and 16 iterations

The radioactivity concentration curves for selected three sets out of the six acrylic resolution rods are shown in Figure 7 for two reconstruction types, OSEM with and without the PSF. The curves are obtained by concentric VOI sampling of varying number of rings depending on the size of the rods—the smallest rods are more densely packed limiting the spread of the sampling rings (Figure 4 *A*). The axial thickness of the sampling rings is the same as for the faceplate sampling, that is, 1 cm, with the sampling occurring in the midpoint of the axial extension of the rods (see marked plane 10 in Figure 2a). Both reconstructions are based on the first scan without any activity outside the FOV. The presented results are for rods #1, #3, and #6 of diameter 4.8 mm (the smallest) and 7.9 and 12.97 mm (the largest), respectively. The rod borders for each set are represented with gray background in both plots. A range of 10 iterations, {2,4,⋯,20}, are shown for both reconstruction types with color ranging from gray to black, respectively. Also, the reconstructed images with 4 and 16 iterations for both reconstruction types are superimposed on both plots in Figure 7.

**FIGURE 8:**
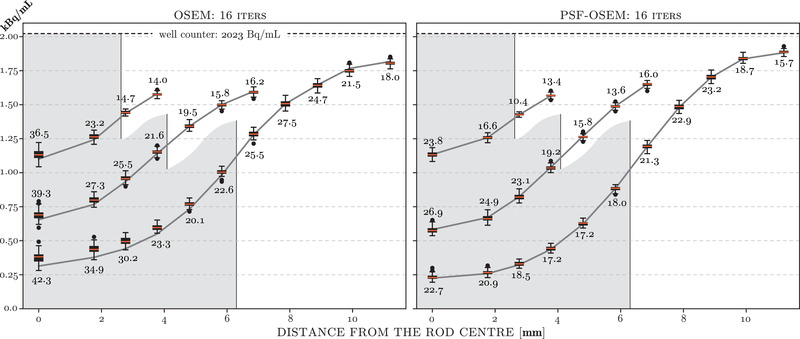
Radioactivity concentration curves for the three sets of acrylic rods as in Figure 7 but only for 16 iterations of OSEM but including boxplots of 50 bootstrap realizations for each concentric ROI ring. The standard errors (SE) in Bq/mL are given for each boxplot

The uncertainty of the radioactivity concentration for each concentric rod VOI and for both types of reconstruction (as above in Figure 7) was analyzed using the bootstrap realizations. The resulting boxplots of uncertainties for 16 iterations of OSEM were superimposed on the concentration curves as shown in Figure 8 for both types of reconstruction. The standard error (SE) values of the radioactivity concentrations are given next to each boxplot in Bq/mL. Importantly, to prove that the two‐stage image registration, necessary for attenuation correction and analysis of the phantom, is robust over the noisy bootstrap realizations, the uncertainty analysis was first performed using static sampling VOIs based on the registration of the sampling templates to the original PET image (without bootstrap resampling). Such generated uncertainties were then compared to the uncertainties generated by not only the PET noise but also by the varying registration of the sampling templates due to the noisy bootstrap realizations. It was found that the uncertainties for both above cases were statistically indifferent, indicating that the image registration does not significantly contribute to the observed uncertainties, and hence, it can be deemed robust.

### 3.3 Quantitative resolution and contrast metrics

The quantitative analysis of the image resolution was possible with the mean concentrations extracted using ring VOIs, to which the error function erf(x) was fit to estimate the knife‐edge response K(x), which can then be differentiated to obtain a Gaussian representing the estimated effective PSF for each rod and insert. The detailed results of fitting and estimating the PSF on selected rod and cold and hot inserts are given in the Supporting Information (Section S3).

**FIGURE 9:**
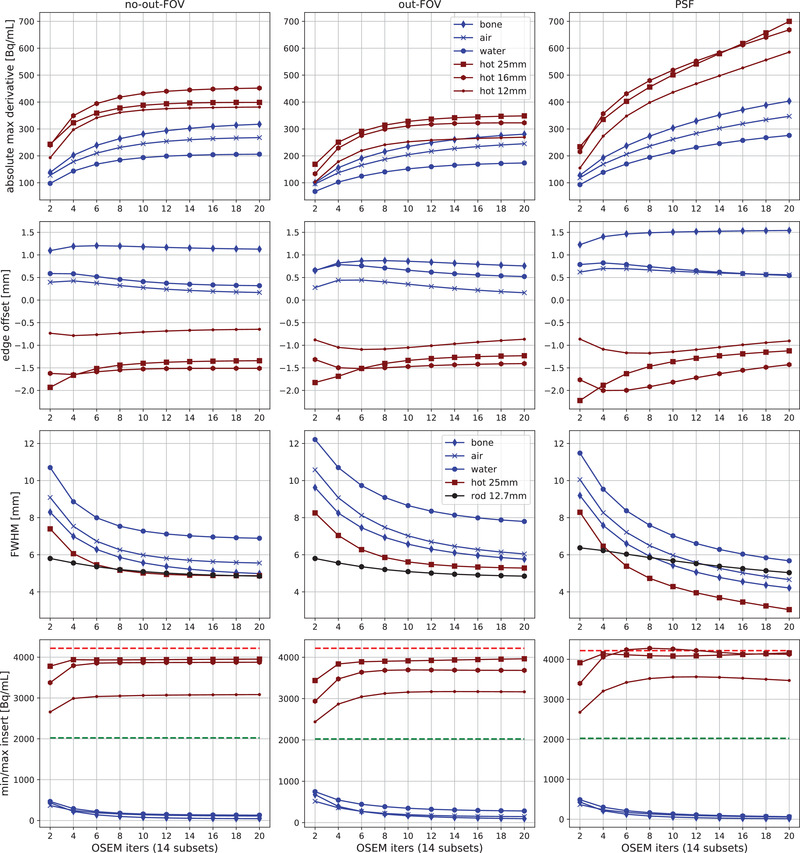
Quantification of spatial resolution and contrast recovery for images of faceplate inserts and largest resolution rod, reconstructed with variable number of OSEM iterations and for acquisition without (left column) and with (middle column) activity outside the FOV, as well with the PSF included in the reconstruction without the activity outside the FOV (right column). The *top three rows* correspond to the resolution metrics: the absolute maximum derivative of K(x), the offset of real and predicted insert boundary, and the FWHM found only for the big insert and the largest rod. The *bottom row* corresponds to the absolute contrast recovered in cold and hot inserts relative to the hot and background activities shown in dashed red and green lines, respectively

By analyzing the knife‐edge response K(x) and its derivative, we extracted four metrics as shown over four rows of plots in Figure 9 for three types of reconstructions shown in the three column plots, that is, reconstructions without and with the activity outside the FOV as well as reconstruction with the PSF. Three of the four metrics describe the spatial resolution, and are the absolute maximum derivative of K(x), that is, the amplitude of the resulting Gaussian representing the effective PSF and the rate of transition from the insert to the background activity concentration at the insert borders. From the top row plots (Figure 9), it can be observed that the hot regions have higher rates than cold regions for all types of reconstruction. The activity outside the FOV reduced the rate of transition, while adding the PSF in the reconstruction significantly improved the rates, especially for the hot inserts. The same is also reflected in the estimated FWHM of the PSF for the large inserts, for which a wide enough sampling exists, as shown in the third row (for reference purposes, we have also shown the estimated FWHM of the largest resolution rod, which is not affected by the outside FOV activity being placed on the other side of the FOV).

The second row of Figure 9 represents the offset from the real to the estimated insert boundaries. The boundaries were estimated from the image using the inflection point of K(x), thus testing the capability of the reconstructed image to predict the insert boundaries. All of the offsets are within the reconstructed image voxel size (<2 mm); however, the offset was slightly larger for PSF reconstruction. The bottom row plots show the absolute contrast (maximum ring values for hot inserts and minimum ring values for cold inserts) achieved for all the reconstructions, with the dashed lines showing the well counter measurements for hot and background concentrations. The activity outside the FOV increased the activity reconstructed in the cold regions, especially for the water insert, while the PSF reconstruction improved the reconstructed concentrations for hot and cold inserts.

**FIGURE 10:**
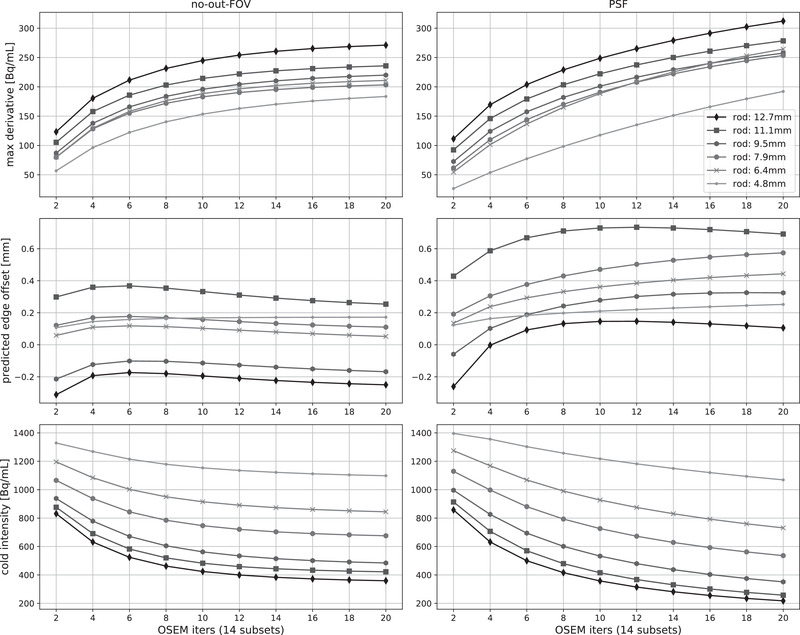
Quantification of spatial resolution and contrast recovery of images of resolution rods reconstructed with variable number of OSEM iterations with (left column) and without (right column) the PSF included in the reconstruction (right column). Three metrics are presented in the three rows from top to bottom: maximum derivative of the knife‐edge response K(x), the offset of the real and predicted insert edges, and the minimum values in the cold regions

A very similar analysis was performed for the resolution rods in Figure 10, showing the same metrics as above, that is, absolute maximum derivative, offset of the predicted insert edges, and minimum reconstructed mean ring VOI values for the cold regions. The FWHM of the estimated PSF has not been plotted for the rods as, apart from the largest, the rods are too densely populated and not enough space is given for wide enough sampling. However, the maximum rate of transition is well reflective of the achieved resolution, which was the best for the largest rods and worst for the smallest rods. The predicted offset between the real and estimated insert edges was significantly below 1 mm. The PSF significantly improved the resolution and contrast (shown in the bottom row plots), but required more OSEM iterations, especially for the smaller rods.

**FIGURE 11:**
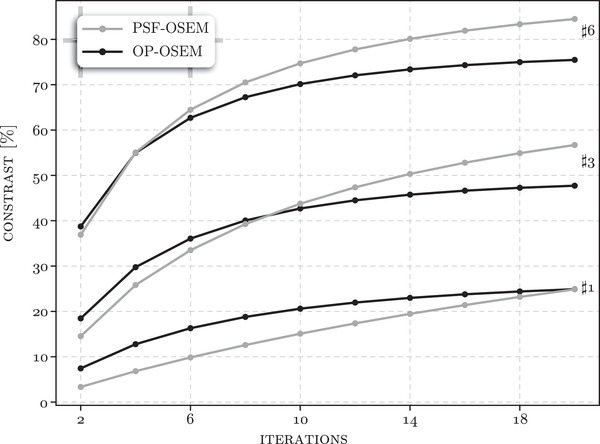
Percentage contrast recovery for the three sections of rods (#1, #3, and #6, cf. Figure 7) and for the OSEM and PSF‐OSEM image reconstructions with iterations varying from 2 to 16. All results are shown for the scan without the activity outside the FOV

Since the gold standard contrast is known based on the well counter measurement, it was possible to plot contrast recovery (calculated as reconstructed concentration/measured by the well counter) in Figure 11 for the three sets of rods and both reconstruction types over the range of iterations examined, that is, {2,4,⋯,20}.

## 4 DISCUSSION

### 4.1 Standard analysis

The primary aim of the proposed software for automated analysis of the ACR phantom is to provide robust and expected results of the most common metrics of SUV and contrast analysis, thus avoiding unreliable and time‐consuming visual interpretations. The choice of image analysis methods can have a significant impact on the evaluation and standardization of PET systems^3^; this includes the size and location of ROIs/VOIs as well as the statistical metrics describing image features such as noise, resolution, and contrast.^1^, ^2^, ^5^, ^8^ Therefore, we have closely followed the SUV worksheet provided by the ACR accreditation for the standard analysis presented in the Supporting Information (Section S1), where we demonstrated the software passing all the ACR criteria for SUV values. The results from this analysis are also in agreement with previously published work on PET data^15^ and on resolution rods when applied to SPECT.^14^, ^16^

### 4.2 Proposed advances

Another important aim of this work was to provide an automated analysis of the ACR phantom, which goes beyond the currently offered automated analyses of the phantom^14^, ^15^, ^16^ in that, it extends the automated analysis for PET/MR scanners as well as offers quantitative metrics, derived in a novel way, to evaluate the spatial resolution for different contrasts and shapes of the inserts. Obtaining a μ‐map representative of the attenuating object for accurate attenuation correction in PET/MR scanners is difficult; hence, the μ‐map in this work is automatically generated from designed templates for any possible configuration of the resolution rods relative to the rest of the phantom. The hardware μ‐maps of the bed and optional head coils are provided by the vendor and accounted for in the reconstruction.

The proposed technique for evaluation of the spatial resolution is based on sampling the images using concentric VOI rings to measure the knife‐edge response function for any insert, hot and cold, provided that they are cylindrical in shape. The knife‐edge response can also be extracted by scanning a uniform cylindrical phantom placed at an angle relative to the z‐axis,^20^ having the advantage of estimating the PSF in the transaxial and axial planes; however, the sampling precision is subject to the placement of the phantom. The limitation of our work lies in that it can estimate the effective PSF in one plane only, which is perpendicular to the long axes of the cylindrical inserts. This can be alleviated by performing two consecutive scans with two different placements of the phantom. Another way of solving it would be by adding the spheres of different sizes^31^ that are used in SPECT image evaluation with the same phantom (the ACR, however, specifically requests the spheres to be removed for PET evaluation/accreditation).

However, the important advantage of using the ACR phantom with the cylindrical inserts is that it can estimate the effective PSF with higher statistical confidence, as cylindrical inserts tend to have greater edge surface compared to spherical inserts. Also, the different inserts simulating water, air, bone, and hot lesions are of value in the application of brain imaging, for example, in dementia and epilepsy, where cold regions of hypometabolism or low amyloid deposition are often encountered. Hence, estimating the spatial resolution as well as contrast and noise at the same time for such regions can provide greater insight into evaluation of different PET/MR and PET/CT scanners. Although it can be argued for the use of printed phantom,^10^, ^17^, ^32^ which are more successful in mimicking the complexity and shapes of living tissue, phantoms with simple spherical or cylindrical shapes can nevertheless provide more insightful and robust evaluation of image quality.

### 4.3 Importance of robust image registration

For the automated methods to work correctly, the phantom components have to be accurately identified. This is even more important for PET/MR applications, for which the μ‐map has to be generated and accurately aligned. This can be done by specifically positioning the phantom during the acquisition^14^, ^16^; using activity profiles and center of gravity;^14^ automatically detecting and locating the insert landmarks,^1^ or by registering predefined templates to the PET image space.^15^ Although these automated methods are potentially more reliable (as shown for multiple retrospective acquisitions^15^), they are particularly challenging when phantoms contain movable components, which was addressed in this work by the two‐stage registration. It has to be noted that rigid‐body image registration of phantom data can be more challenging as it is more ill‐conditioned (with many more potential local minima in the optimization process) than, for example, rigid‐body registration of the human head. Nevertheless, with the specifically designed registration templates, we were able to achieve robust registration, which was assessed by running independent registrations for each noisy bootstrap PET image realization, finding that the registration does not produce detectable uncertainty above the intrinsic PET noise. Furthermore, based on previous research^30^ and the visual inspection of registration, it can be surmized that more precise registration is also likely to be more accurate.

### 4.4 Resolution and contrast for different image reconstructions

The observed rates of the radioactivity concentration changes when transitioning between cold and hot regions, and as measured by the maximum derivative of the knife‐edge response, are representative not only of the achieved image contrast but also of the image resolution, which were variable for different inserts. For example, the hot inserts tend to have higher rates of transitioning between regions and achieving higher spatial resolution. For the PSF reconstruction of the largest hot insert, the estimated effective spatial resolution was below 4 mm, which is likely to be the result of Gibbs artifacts.

The cold faceplate inserts were observed to behave differently to the cold rods in terms of the estimated resolution, especially for the early OSEM iterations. This may be caused by the different axial position of the rods (more central with greater scanner sensitivity) and/or different size and attenuation properties of the inserts. The added activity outside the FOV reduced the contrast particularly for cold inserts in the faceplate and reduced the resolution for all inserts (the FWHM of the measured PSF was wider by 1−2 mm). Hence, measuring image resolution using different inserts, at different locations, within an attenuating medium, may be more representative to human scans compared to the measurements of point sources or uniform phantoms to estimate the resolution.

The reconstruction of cold areas within a warm background is known to be challenging and was observed for the water insert with higher reconstructed concentration than any other cold regions, likely due to the scanner table proximity with more events attenuated and scattered by the table compared to the two higher positioned air and bone inserts. This seems further corroborated by the higher detected noise in the cold region as observed by the bootstrap uncertainty analysis. Including the PSF in the reconstruction of the resolution rods benefits from higher contrast and resolution; however, the algorithm needs more OSEM iterations, particularly for the smaller rods needing more iterations to see improved contrast.

Note that the provided reference measurement of the radioactivity concentration for the background and hot inserts was measured using the well counter, which was cross‐calibrated with the scanner reconstruction and not the *NiftyPET* reconstruction. Nevertheless, in principle, we were expecting the calibration to be in greater agreement with the reconstructed concentration; this, however, did not impact the presented methodology and software. *NiftyPET*, however, allowed easy modification of reconstruction parameters and straightforward implementation of custom μ‐maps.

### 4.5 Computational resources

The full processing chain of generating 50 bootstrap realizations for a single‐image reconstruction type, followed by upsampling to high‐resolution grid and the two‐stage image registration for each realization, takes approximately 4 h (12 h for the three reconstructions presented here) using Intel i9‐9820X CPU @ 3.30 GHz with 128 GB of RAM and the NVIDIA TITAN RTX GPU running on CUDA version 10.2. To perform the uncertainty analysis, this automated process would need to be repeated for any other PET acquisition; however, the uncertainty analysis will likely be representative for ACR phantom acquisitions scanned on a similar scanner with similar radioactivity dose.

### 4.6 Application and future work

The presented software for automated analysis is intended to be used in the characterization of eight PET/MR scanners across the Dementias Platform UK (DPUK) network, and to facilitate advanced analysis of the brain by accounting for the scanner differences (five scanners have the time‐of‐flight technology; the presented data came from nontime‐of‐flight scanner). The presented data and software will also be used in the research of novel imaging methods as part of the collaborative computational project (CCP) in synergistic image reconstruction for biomedical imaging, https://www.ccppetmr.ac.uk.

## 5 CONCLUSION

The presented software platform for automated ACR PET phantom analysis proposes a novel evaluation of PET/MR and PET/CT imaging systems using high definition sampling VOIs to accurately extract the knife‐edge response and estimate the spatial resolution. This platform also enables quantitative testing and evaluating of novel or existing reconstruction algorithms, when using a single PET acquisition. The acquired PET/MR data, together with the code, are available as open source (see our website at https://nmi.cs.ucl.ac.uk and the software documentation at https://niftypet.readthedocs.io).

## CONFLICT OF INTEREST

The authors have declared no conflict of interest.

## Supporting information

Supporting Information

Supporting Information

Supporting Information

Supporting Information

Supporting Information

## Footnotes

1 The native PET voxel size is not exactly isotropic; that is, the exact voxel dimensions in x,y,z are 2.08626×2.08626×2.03125 mm${}^{3}$, respectively. The voxel dimensions of the templates are precise.

2
https://accreditationsupport.acr.org/support/solutions/articles/11000062800‐phantom‐testing‐pet‐revised‐11‐30‐2020‐
