# Supplementary material for: Advanced quantitative evaluation of PET systems using the ACR phantom and NiftyPET software
Source: Med Phys. 2022 Mar 31;49(5):3298–313. doi: 10.1002/mp.15596 (PMC9289925; doi:10.1002/mp.15596)
Supplement: Supplementary file 1 — Supporting Information [file MP-49-3298-s004.pdf]

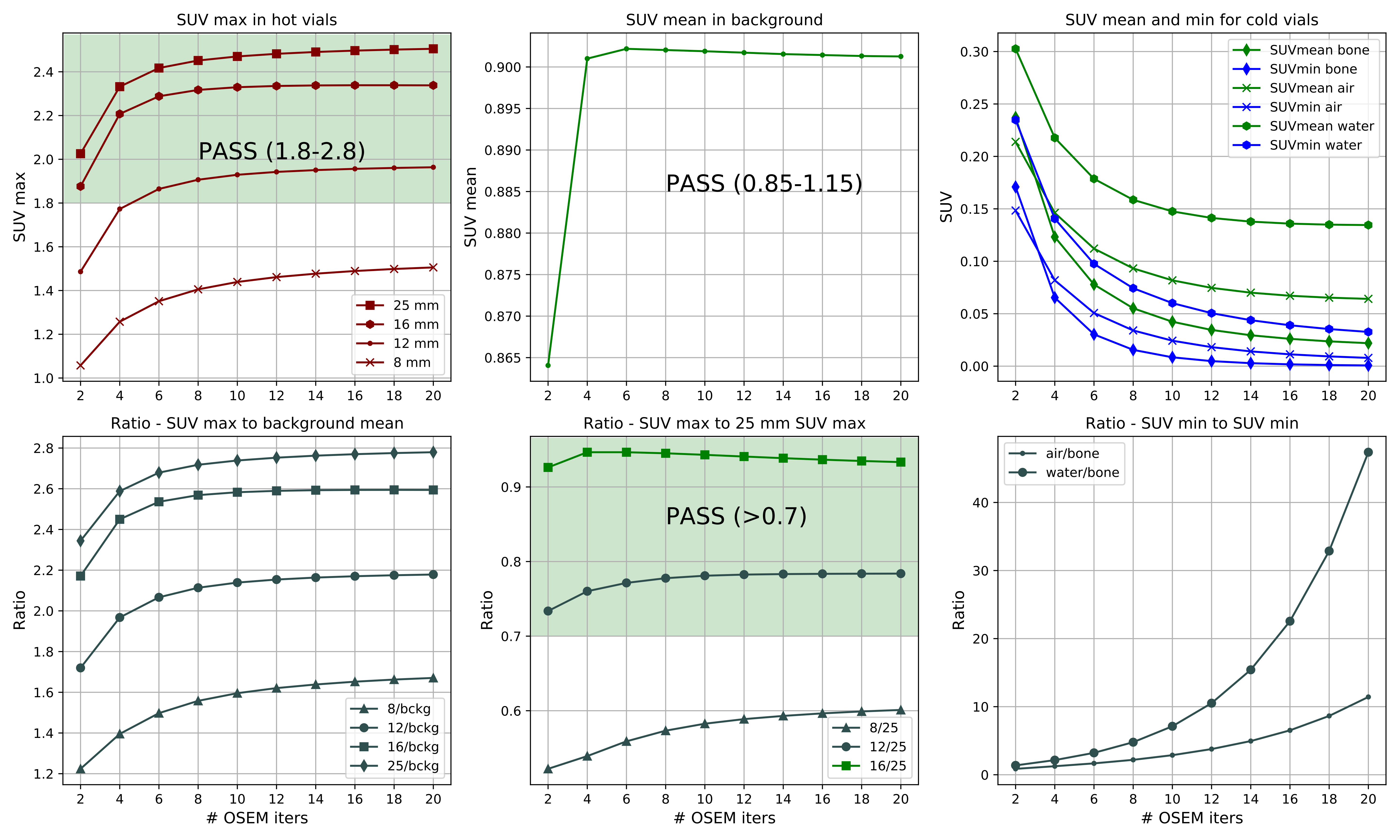
SUV max in hot vials
PASS (1.8-2.8)
SUV max
25 mm
16 mm
12 mm
8 mm
SUV mean in background
PASS (0.85-1.15)
SUV mean
SUV mean and min for cold vials
SUVmean bone
SUVmin bone
SUVmean air
SUVmin air
SUVmean water
SUVmin water
SUV
Ratio - SUV max to background mean
Ratio
8/bckg
12/bckg
16/bckg
25/bckg
OSEM iters
Ratio - SUV max to 25 mm SUV max
PASS (>0.7)
Ratio
8/25
12/25
16/25
OSEM iters
Ratio - SUV min to SUV min
air/bone
water/bone
Ratio
OSEM iters
