# Supplementary figures and images for: Advanced quantitative evaluation of PET systems using the ACR phantom and NiftyPET software
Source: Med Phys. 2022 Mar 31;49(5):3298–313. doi: 10.1002/mp.15596 (PMC9289925; doi:10.1002/mp.15596)

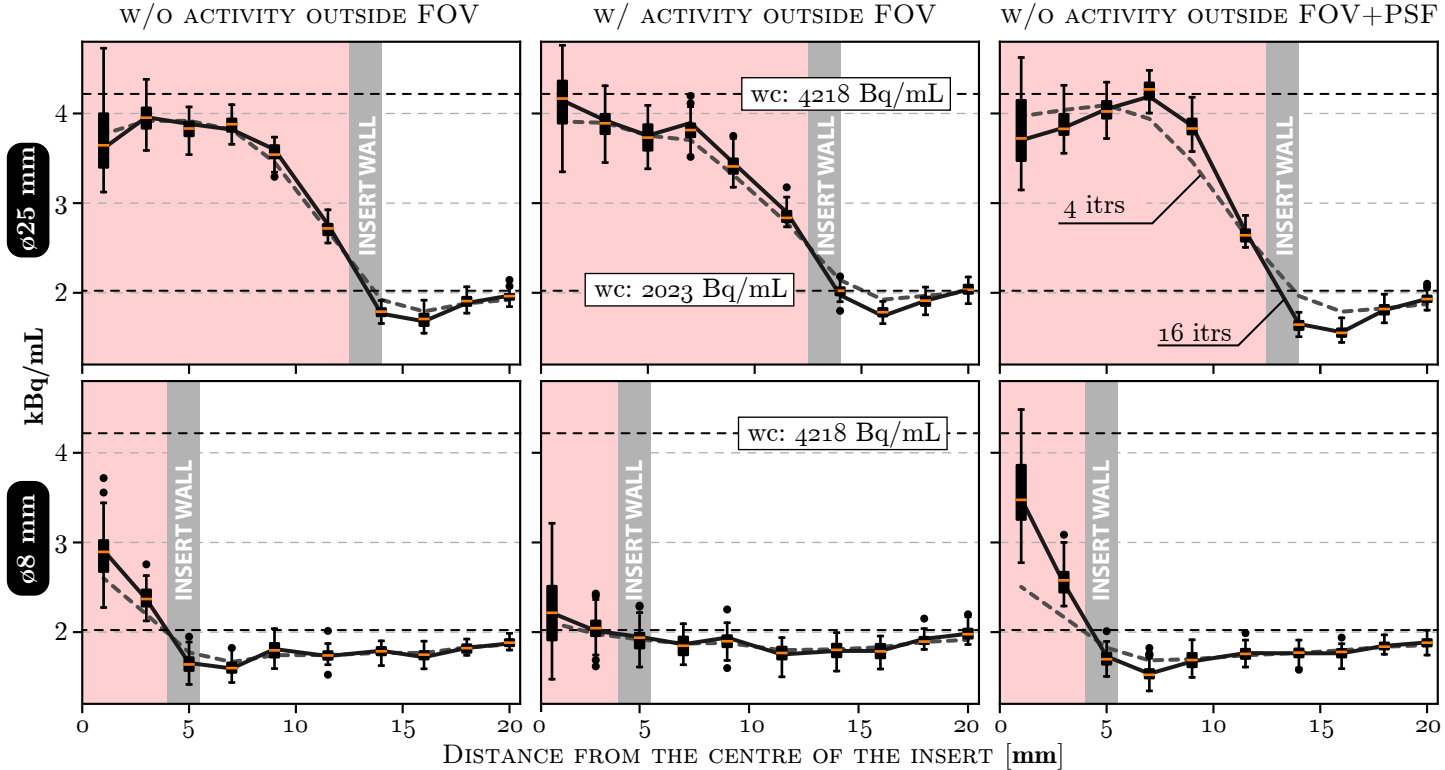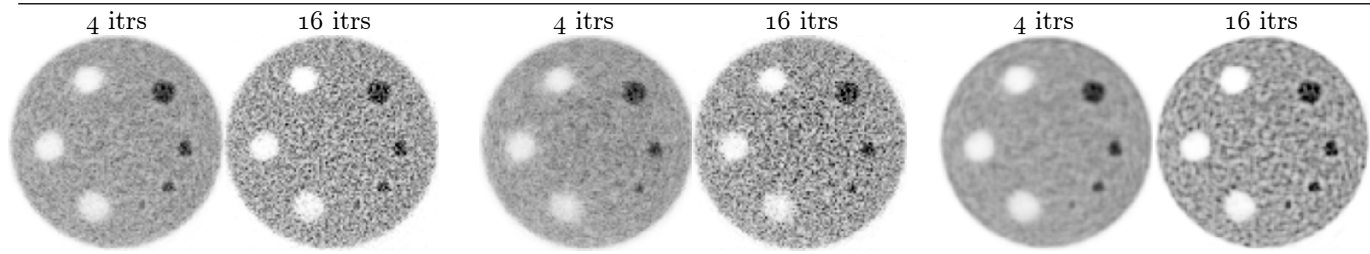

Supplement: Supplementary file 2 — Supporting Information [file MP-49-3298-s002.pdf]

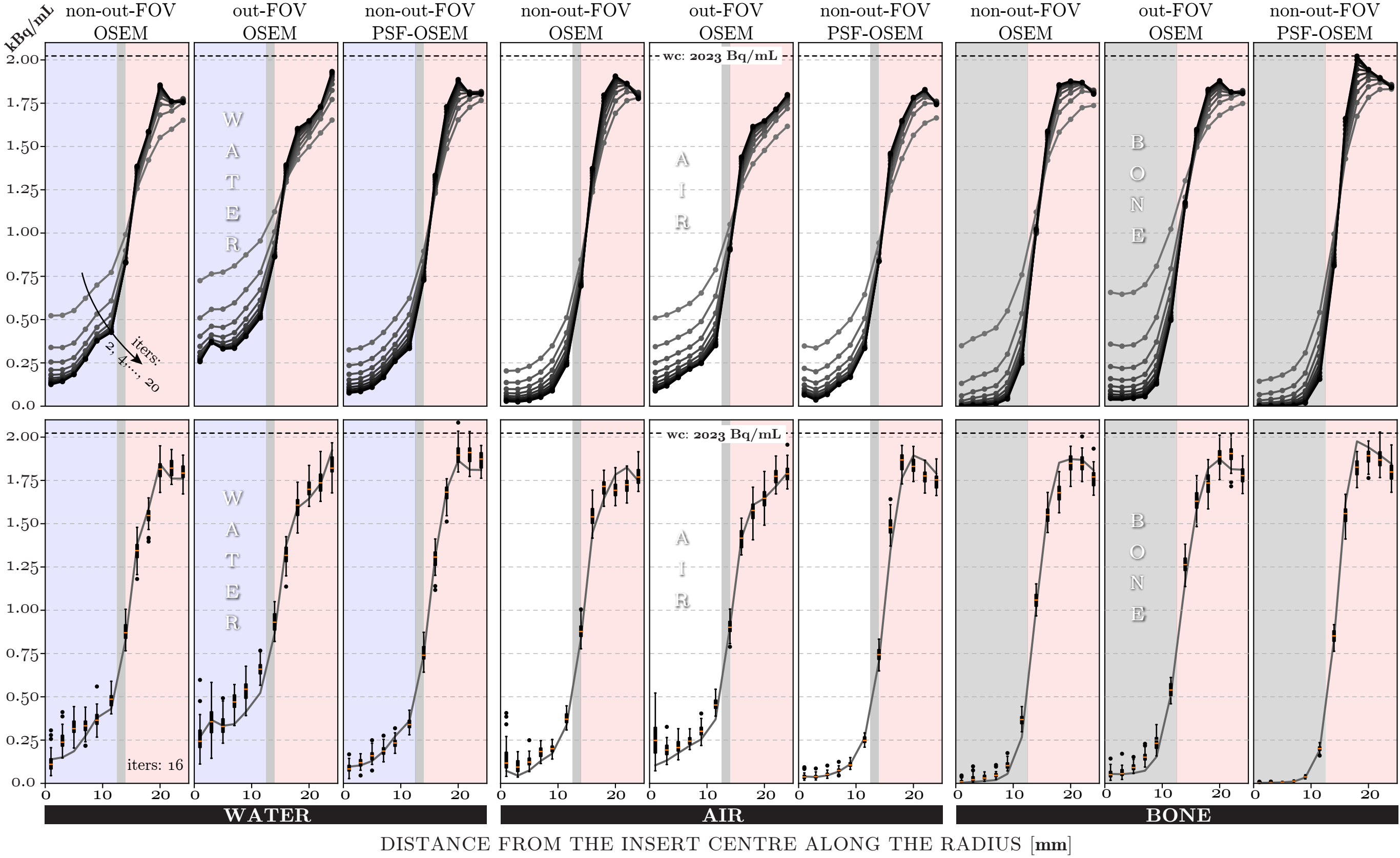

Supplement: Supplementary file 3 — Supporting Information [file MP-49-3298-s001.pdf]

Fitting erf; OSEM with 8i/14s

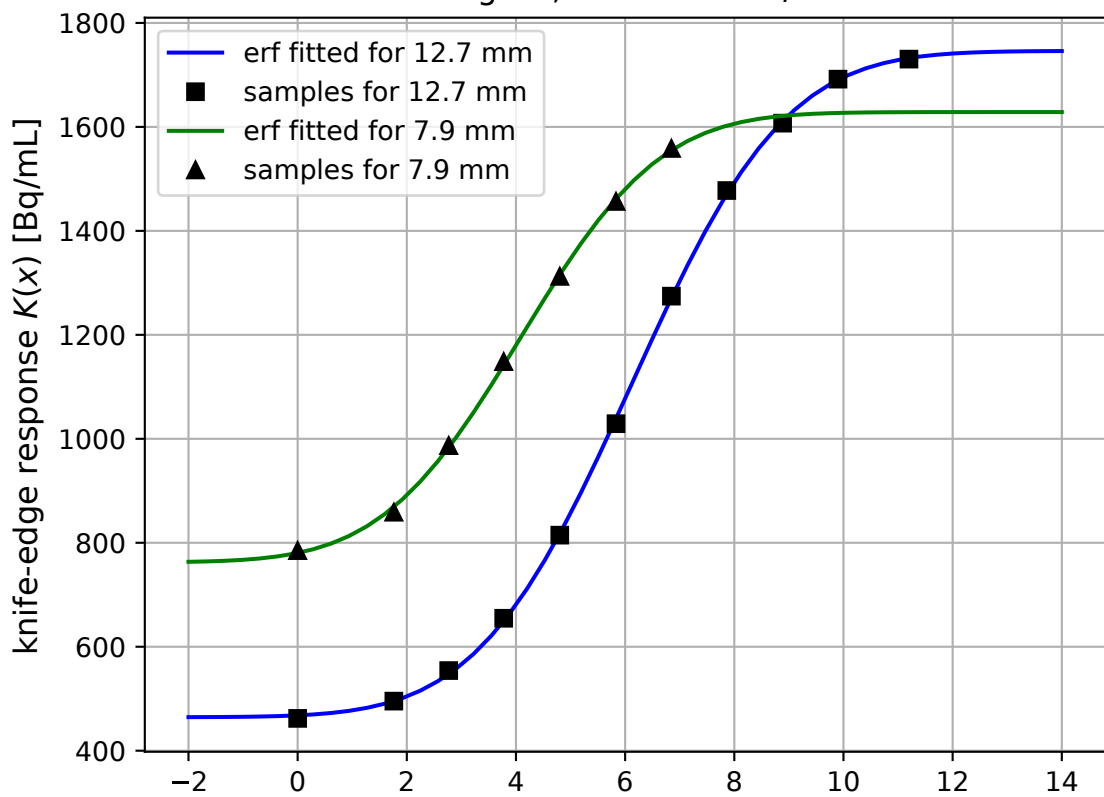

Fitting erf; OSEM with 8i/14s

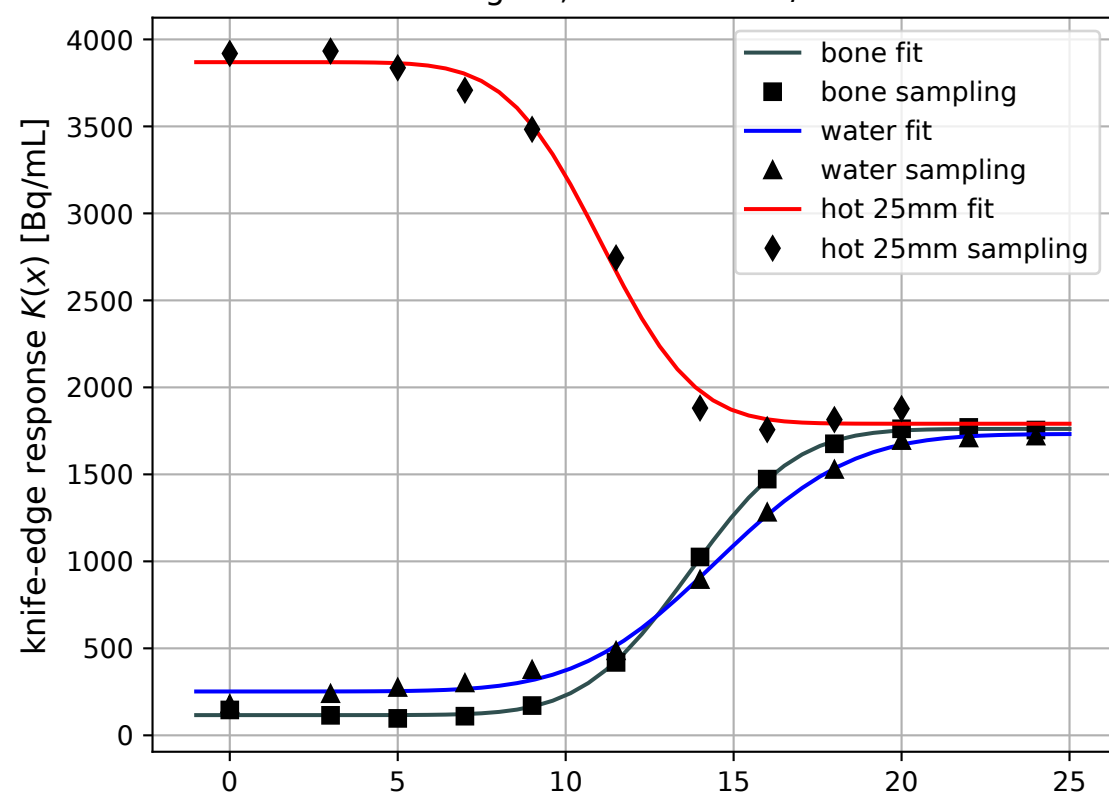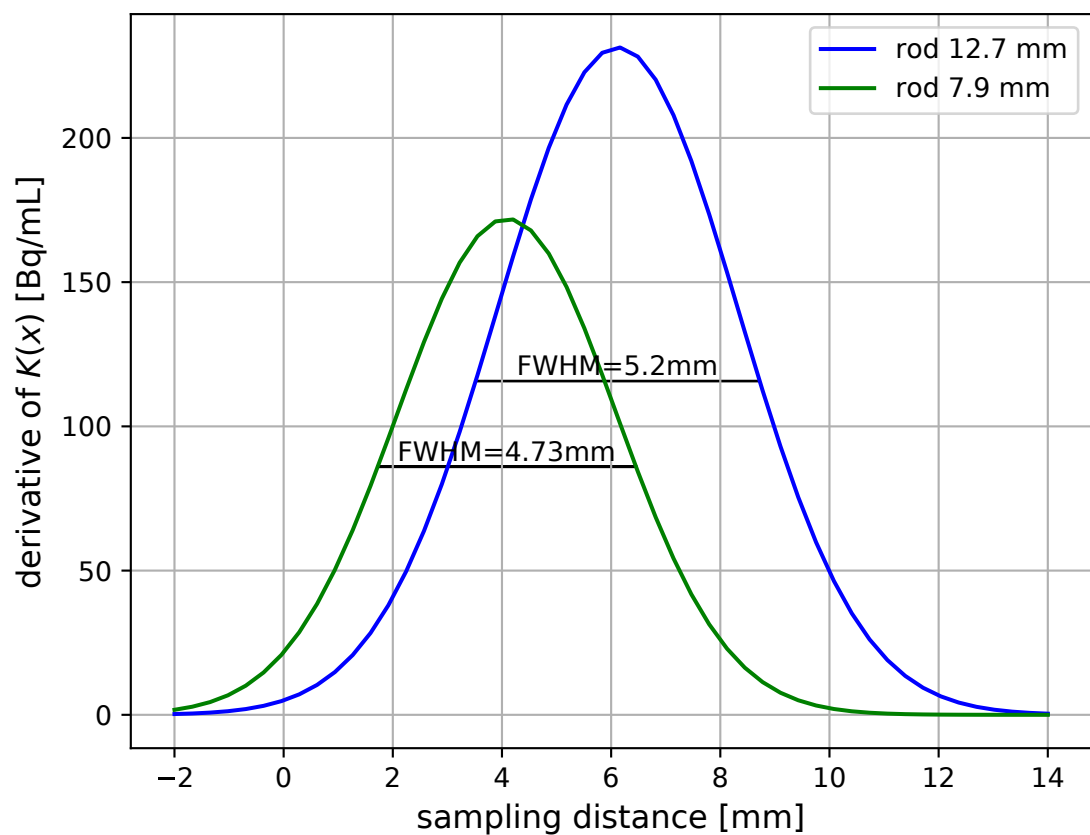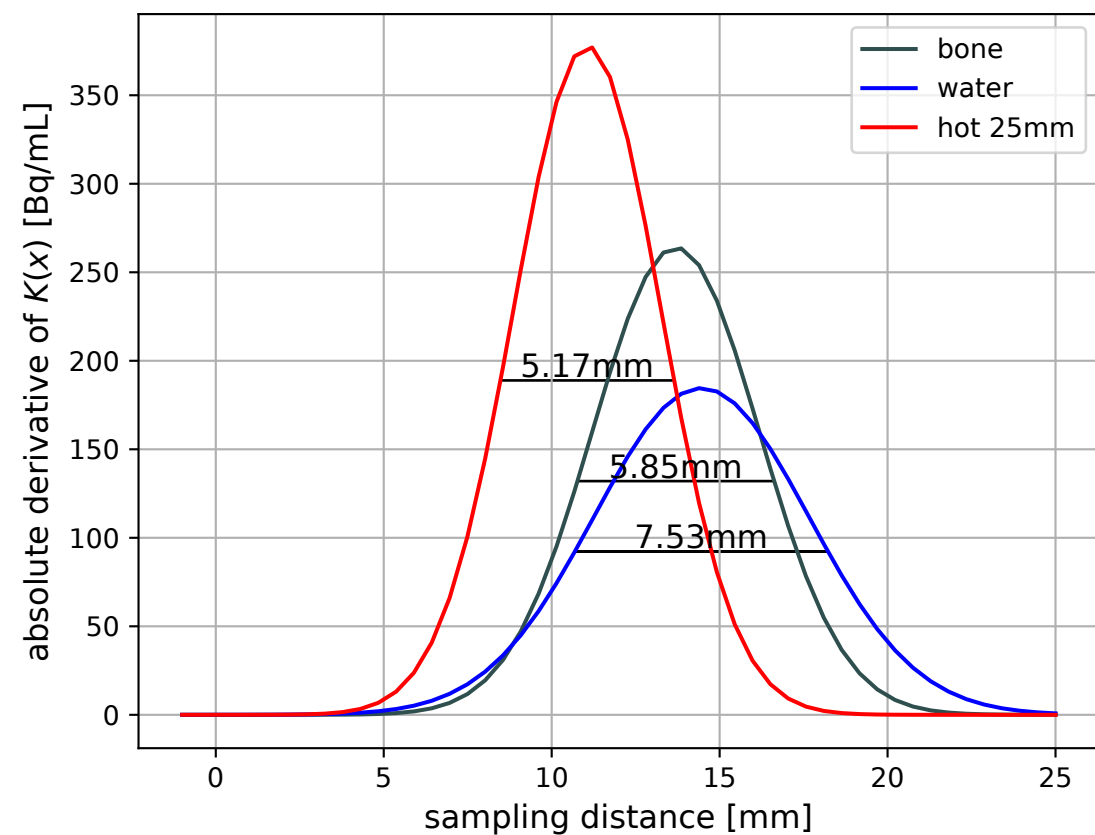

Supplement: Supplementary file 4 — Supporting Information [file MP-49-3298-s005.pdf]
